# Editorial: The role of dietary interventions in the regulation of host-microbe interactions: Volume II

**DOI:** 10.3389/fcimb.2022.1078271

**Published:** 2022-11-11

**Authors:** Qingyao Shang, Yuan Gao, Huaijun Tu, Tingtao Chen

**Affiliations:** ^1^ Departments of Geriatrics, the Second Affiliated Hospital of Nanchang University, Nanchang, Jiangxi, China; ^2^ National Cancer Center/National Clinical Research Center for Cancer/Cancer Hospital, Chinese Academy of Medical Sciences and Peking Union Medical College, Beijing, China; ^3^ The Institute of Translational Medicine, Nanchang University, Nanchang, Jiangxi, China; ^4^ Beijing Neurosurgical Institute, Capital Medical University, Beijing, China

**Keywords:** dietary interventions, intestinal microbiota homeostasis, dysbiosis, probiotics/prebiotics, neurodegenerative disease, host health

Intestinal microbiota, a complex ecosystem of microbes that inhabits and critically maintains homeostasis of the gastrointestinal tract, exerts a major impact on host physiological, nutritional and immunological processes. Among the multiple factors related with intestinal microbiota, diet is regarded as the most important determinants. Dietary intervention mainly regulates the composition of intestinal microbiota by ingesting probiotics/prebiotics, dietary fiber, etc., and then improves host immunity, metabolic processes and nutrients bioavailability, inhibits oxidative stress and inflammatory pathways to achieve the purpose of improving host health. Therefore, in the present Research Topic, we have collected eleven articles related to the regulation of intestinal microbiota and host health through dietary interventions, including nine original research articles, one prospective clinical study and one review in the fields of human systemic diseases (allergy, hyperuricemia, hyperlipidemic), gastrointestinal diseases (colorectal polyps), neurodegenerative diseases (Alzheimer’s disease (AD), Parkinson’s disease (PD)), and animal husbandry (calves, broiler feed, weaned piglets, chickens).

Systemic diseases are diseases that affect all organs and systems of the body, which are often chronic diseases such as allergy and diabetes. Intestinal microbiota plays an important role in the development of systemic diseases. Based on β-lactoglobulin-induced allergy mice model, Tian et al. explored the mechanism of *Bifidobacterium animalis* KV9 (KV9) and *Lactobacillus vaginalis* FN3 (FN3) on alleviating allergic reactions and regulating immune cell function. KV9 and FN3 intervention activated the toll-like receptor 4-NF-kB signaling pathway in intestinal dendritic cells, resulting in an increase in interleukin-12 secretion and a decrease in interleukin-4 secretion, which have the potential to promote T-cell differentiation into T helper type 1 cells.

Hyperuricemia is a systemic disease in which excess uric acid (UA) is present in the blood, increasing risk of chronic kidney disease and gout. Using a hyperuricemia mouse model, Cao et al. examined the impact of probiotics - *L. paracasei* X11 on UA metabolism. By correcting the proportion of *Bacteroidetes* to *Firmicutes* to promote the intestinal microbiota homeostasis, *L. paracasei* inhibited the renal pro-inflammatory cytokine IL-1, restored normal levels of hepatic metabolic enzymes (adenosine deaminase, xanthine oxidase), transporter protein expression (GLUT9, NPT1, and URAT1), and lowered serum UA by 52.45%. In addition, prebiotics and probiotics can reduce metabolic syndrome by regulating intestinal microbiota. Verified by *in vitro* screening, Pi et al. found that oligosaccharide as a prebiotic improved the growth of live combined *Enterococcus faecium* and *Bacillus subtilis* (LCBE). Based on a hyperlipidemic mouse model, LCBE combined with oligosaccharide diet significantly reduced plasma cholesterol levels, lowered the *Firmicutes/Bacteroidetes* ratio and increased the relative abundance of *Akkermansia* and *Bifidobacteria*, which was proven to help avoid functional gastrointestinal disorders.

Gastrointestinal disorders are diseases of the human digestive system, closely related to the disruption of intestinal microbiota homeostasis, and therefore are the main targets of dietary interventions. Pan et al. characterized the distribution and diversity of mucin-degrading bacteria in the human gut. Mucin-degrading bacteria were widely distributed in human intestinal, mainly *Bacteroides spp*, which reduced the inflammatory response brought on by *E. coli* by inhibiting the NF-κB pathway and enhanced the epithelial tight junction. In another prospective clinical study led by Liu et al., patients after intestinal polypectomy received oral *B. animalis* MH-2 to assess its effect on postoperative symptoms (pain, bloating, difficult defecation). The results showed that MH-2 helped restore intestinal microbiota diversity by increasing the relative abundance of *Bifidobacterium*, while decreasing the relative abundance of *Clostridium* spp., thereby alleviated difficult defecation and shortened recovery time.

Neurodegenerative diseases, like AD, are debilitating, progressive, neurodegenerative conditions, which directly associated with the dysbiosis of intestinal microbiota. Using an amyloid-β-induced AD mouse model, environmental enrichment (EE) training together with the *B. breve* CCFM1025 intervention were examined by Zhu et al. for their ability to reduce neuroinflammation and cognitive impairment. Uptake of EE+*B. breve* CCFM1025 dramatically raised *B. longum*’s relative abundance, decreased *B. pseudocatenulatum*’s relative abundance, and reduced 5-hydroxyindole acetic acid levels, which can mimic the composition of healthy brain and improve cognitive performance.

Beyond the application in human health, dietary interventions also play an important role in animal husbandry. As a common disease in livestock, diarrhoea caused by pathogenic *Escherichia coli* can disrupt the intestinal barrier in newborn calves. He et al. established calf diarrhea model by taking pathogenic *E. coli* O1 orally to inhibit the proliferation of probiotic bacteria, such as *Butyricoccus* and *Lactobacillus*, which significantly increased serum IL-6 level, indicating impaired intestinal barrier function and immune function. In addition, Cao et al. proposed an effective solution-dietary addition of potassium magnesium sulfateon (PMS). PMS significantly increased the abundance of intestinal microbiota, especially *Ruminococcaceae* and *Peptostreptococcaceae*, and inhibited interleukin-1β level and promoted IgM level, which enhanced the antioxidant capacity and modify intestinal immunity of weaned piglets.

To reduce the impact of dietary sources of pathogenic bacteria and toxins, Wang et al. added activated charcoal-herb extractum complex (CHC) to broiler feed. CHC intake increased the abundance of probiotic bacteria (*Romboutsia* and *Lactobacillus*) and reduced the abundance of the pathogenic bacteria (*Alistipes*), which in turn reduced serum levels of interleukin-1β and interferon-γ, exhibiting beneficial effects on immune status and intestinal microbiota composition. In addition, Amevor et al. proposed that the addition of quercetin and vitamin E to chicken feed could promote egg production and immunity. Quercetin partially abrogated the disruption of the intestinal microbiota by increasing *Lactobacillus* abundance and decreasing *Ruminicoccaceae* abundance. Vitamin E supplementation increased *Rikenellaceae*, which promoted fermentation of glucose, mannose and lactose to form intestinal-beneficial organic acids.

PD is a progressive, degenerative disorder that affects 10 million people worldwide. Zhu et al. reviewed recent studies related to the role of intestinal microbiota on the development of PD. Dysbiotic intestinal microbiota can increase intestinal permeability, worsened neuroinflammation, abnormal aggregation of α-synuclein fibrils, oxidative stress, and reduced neurotransmitter production. Dietary intervention based on probiotics/prebiotics can alter the composition of the intestinal microbiota and modulate the microbiota-gut-brain axis for the PD treatment.

In conclusion, this Research Topic provides readers with an overview of the impact of dietary intervention on the regulation of intestinal microbiota and host health, further elucidating the interaction between diet and intestinal microbiota. However, there are still many difficulties in achieving precision medical therapy through dietary interventions until the physiological and molecular mechanisms behind can be elucidated in depth. In order to address these issues, multi-omics technologies such as microbiomics, metabolomics and proteomics should be jointly utilized to study the detailed relationship between intestinal microbiota and diet and related diseases.

## Author contributions

QS, YG, and HT wrote this article, TC and HT revised this article. All authors made a substantial, direct, and intellectual contribution to this work and approved it for publication.

## Funding

This work is supported by grants from the National Natural Science Foundation of China (Grant no. 82060638), and Double thousand plan of Jiangxi Province (high end Talents Project of scientific and technological innovation).

## Acknowledgments

We greatly appreciate the contributions to this Research Topic by all authors and reviewers. We also thank all the guest associated editors of the Research Topic and the editorial board of the journal of *Frontiers in Cellular and Infection Microbiology*, for their support.

## Conflict of interest

The authors declare that the research was conducted in the absence of any commercial or financial relationships that could be construed as a potential conflict of interest.

## Publisher’s note

All claims expressed in this article are solely those of the authors and do not necessarily represent those of their affiliated organizations, or those of the publisher, the editors and the reviewers. Any product that may be evaluated in this article, or claim that may be made by its manufacturer, is not guaranteed or endorsed by the publisher.

